# Transcriptomic signatures of cold acclimated adipocytes reveal CXCL12 as a Brown autocrine and paracrine chemokine

**DOI:** 10.1016/j.molmet.2025.102102

**Published:** 2025-01-21

**Authors:** Marina Agueda-Oyarzabal, Marie S. Isidor, Kaja Plucińska, Lars R. Ingerslev, Oksana Dmytriyeva, Patricia S.S. Petersen, Sara Laftih, Axel B. Pontoppidan, Jo B. Henningsen, Kaja Rupar, Erin L. Brown, Thue W. Schwartz, Romain Barrès, Zachary Gerhart-Hines, Camilla C. Schéele, Brice Emanuelli

**Affiliations:** 1 Novo Nordisk Foundation Center for Basic Metabolic Research, Faculty of Health and Medical Sciences, University of Copenhagen, Copenhagen, Denmark; 2 Institut de Pharmacologie Moléculaire et Cellulaire, Université Côte d’Azur & Centre National pour la Recherche Scientifique (CNRS), Valbonne, 06560, France

**Keywords:** Brown adipocyte, Secretome, CXCL12, Cold acclimation, Intercellular communication

## Abstract

Besides its thermogenic capacity, brown adipose tissue (BAT) performs important secretory functions that regulate metabolism. However, the BAT microenvironment and factors involved in BAT homeostasis and adaptation to cold remain poorly characterized. We therefore aimed to study brown adipocyte-derived secreted factors that may be involved in adipocyte function and/or may orchestrate intercellular communications. For this, mRNA levels in mature adipocytes from mouse adipose depots were assessed using RNA sequencing upon chronic cold acclimation, and bioinformatic analysis was used to identify secreted factors. Among 858 cold-sensitive transcripts in BAT adipocytes were 210 secreted factor-encoding genes, and *Cxcl12* was the top brown adipocyte-enriched cytokine. *Cxcl1*2 mRNA expression analysis by RT-qPCR and fluorescence in situ hybridization specified *Cxcl12* distribution in various cell types, and indicated its enrichment in cold-acclimated brown adipocytes. We found that CXCL12 secretion from BAT was increased after chronic cold, yet its level in plasma remained unchanged, suggesting a local/paracrine function. *Cxcl12* knockdown in mature brown adipocytes impaired thermogenesis, as assessed by norepinephrine (NE)-induced glycerol release and mitochondrial respiration. However, knockdown of *Cxcl12* did not impact β-adrenergic signaling, suggesting that CXCL12 regulates adipocyte function downstream of the β-adrenergic pathway. Moreover, we provide evidence for CXCL12 to exert intercellular cross-talk via its capacity to promote macrophage chemotaxis and neurite outgrowth. Collectively, our results indicate that CXCL12 is a brown adipocyte-enriched, cold-induced secreted factor involved in adipocyte function and the BAT microenvironment communication network.

## Highlights

• Physiological cold acclimation differentially reshapes the adipocyte secretome from distinct depots.
• CXCL12 is enriched in brown adipocytes upon cold acclimation and BAT releases CXCL12 into the extracellular milieu.
• CXCL12 facilitates norepinephrine-stimulated thermogenesis in brown adipocytes.
• CXCL12 induces neurite outgrowth *in vitro*.
• Brown adipocyte-produced CXCL12 mediates chemotaxis with macrophages *in vitro*.

## 1 Introduction

Brown adipose tissue (BAT) produces heat through non-shivering thermogenesis [1], and its activation is associated with improved cardiometabolic health in rodents and humans [[2], [3], [4]]. This makes BAT an attractive target to combat obesity and its associated cardiometabolic complications. However, BAT distribution and activity vary greatly among individuals [4,5], stressing the need for novel strategies to enhance BAT prevalence and activity. Targeting the secretory function of BAT offers a promising approach, as several factors commonly referred to as brown adipokines (or batokines) have been shown to regulate energy expenditure and metabolic health [[6], [7], [8], [9], [10]], underscoring the significance of the endocrine role of BAT in systemic energy balance.

In addition, the heterogeneity of the BAT microenvironment is an important factor contributing to tissue remodeling during cold adaptation [[11], [12], [13]]. Adipocytes, precursor cells, immune cells, sympathetic nerve projections, and endothelial cells orchestrate complex cellular cross-talk by secreting an array of mediators to support tissue adaptation. The release of cytokines, angiogenic, neurotrophic and adipogenic factors stimulated by cold facilitates the accommodation and expansion of thermogenic tissues [13]. In this process, tissue resident macrophages and other immune cells, such as eosinophils and T regulatory cells, impact adipose thermogenic functions through complex networks [[14], [15], [16], [17], [18]]. Thus, albeit there has been some advancement in understanding the BAT microenvironment, further research is needed to characterize adipocyte cross-talk within the tissue's milieu, particularly focusing on the brown-adipocyte derived molecules.

Here, we aimed to identify novel factors by which BAT coordinates the thermogenic response; we therefore utilized deep RNA sequencing of tissue-derived mature adipocytes after prolonged exposure to cold or thermoneutrality. We identified 210 differentially regulated transcripts encoding secreted factors between thermoneutral and cold conditions in BAT-adipocytes, and 16 in inguinal white adipose tissue (iWAT) adipocytes. Among those, *Cxcl12* was the most induced chemokine-encoding gene, primarily upregulated in mature brown adipocytes. In other cell types, CXCL12 –also known as stromal cell-derived factor 1 (SDF-1)- is a well-described CXC family chemokine that binds CXCR4 and CXCR7/ACKR3 and is involved in cell survival, migration, neuronal growth and angiogenesis to facilitate tissue development and homeostasis [19]. CXCL12 also contributes to disease progression, including mediation of obesity-associated inflammation and insulin resistance in white adipose tissue [20,21]. While exogenous administration of recombinant CXCL12 was proposed to activate brown adipocytes *in vitro* and the CXCL12/CXCR4 axis may modulate BAT function in obesity [22,23], a recent study showed that CXCL12 could be produced by smooth muscle cells in BAT to recruit macrophages and support sympathetic input [24]. Yet, the role of brown adipocyte-derived CXCL12 for BAT function and remodeling in response to cold remains largely elusive.

We therefore explored the role of CXCL12 produced by brown adipocytes in response to cold and its potential autocrine and paracrine functions in BAT. Cellular characterization of CXCL12 exposed its requirement for optimal brown adipocyte thermogenic performance. In addition, CXCL12 mediated intercellular communication between brown adipocytes and other cell types *in vitro*, suggesting a pleiotrotopic role for CXCL12 in supporting BAT sustained adaptation to cold.

## 2 Materials and methods

### 2.1 Animals

Mice were tested in accordance with the Danish Animal Experiments Inspectorate (2015-15-0201-00728, 2019-15-0201-00287). All mice were kept at 12-hour light/dark cycles (lights on at 6:00 and off at 18:00) and were given water and a chow diet (Altromin. Cat. 1310). For temperature and fractionation experiments, 10-week old C57Bl/6 male mice were employed as previously described [25,26].

### 2.2 Thermoneutrality and cold exposure

Prior to cold challenge, mice were first acclimated to 29 °C (thermoneutrality, TN) in temperature-controlled incubators (Memmert HPP750Life) for 21 days, and then transferred to 5 °C for 3, 7 or 21 days. Control groups were kept at 29 °C for the corresponding times.

### 2.3 Isolation of adipose tissue cell fractions

Interscapular BAT, inguinal WAT (iWAT) and epididymal WAT (eWAT) were harvested from mice following chronic exposure to cold or TN and 2 depots were merged per sample (final n = 6). Tissues were washed with phosphate buffered saline (PBS) (ThermoFisher Scientific, Cat. 20012-068) supplemented with 10 mM CaCl_2_ (Sigma Aldrich, Cat. 21115) and 0.5% fatty acid-free bovine serum albumin (BSA) (Sigma Aldrich, Cat. A8806), minced and digested with collagenase solution (2 mg/ml Collagenase type II; Sigma Aldrich, Cat. C6885 in PBS) for 30 min at 37 °C. The samples were centrifuged at 800 g for 15 min to obtain mature adipocyte fraction (MAF) and stromal vascular fraction (SVF). Fractions were then washed with PBS and lysed in TRIzol (ThermoFisher Scientific, Cat. 15596026) for RNA extraction.

### 2.4 cDNA library preparation and RNA sequencing

RNA from adipose tissue cell fractions was extracted using a combined TRIzol/chloroform/RNeasy kit (Sigma Aldrich, Cat. C2432; Qiagen, Cat. 74104) spin column protocol as previously described [27]. Integrity of RNA was assessed using RNA 6000 Nano Bioanalyzer chips (Agilent Technologies). RNA concentrations were tested using Qubit (ThermoFisher Scientific) and NanoDrop (ThermoFisher Scientific). Ribosomal RNAs were depleted and mRNA was fragmented to prepare cDNA libraries as previously described [28]. The size and integrity of the libraries were assessed using Bioanalyzer DNA High-Sensitivity Chips (Agilent, Cat. 5067-4626). For RNA sequencing, the samples were pooled and diluted to a concentration of 1.8 – 2pM and sequencing was performed using an Illumina NextSeq 500/500 High-Output V2 Kit (Illumina, Cat. 2002490X) with up to 18 samples sequenced per lane. To monitor the sequencing performance, 1% PhiX spike was added as a control (Illumina, Cat. FC-110-3001).

### 2.5 Bioinformatic analysis

The STAR aligner [29] v. 2.5.3a was used to align RNA-seq read against the mm10 mouse genome assembly and GENCODE vM15 mouse transcripts [30]. The software program featureCounts [31] v. 1.5.3 was used to map reads to genes. Testing for differential expression was performed using edgeR [32] using the quasi-likelihood framework with a fitted model of the form ∼ 0 + group where group encoded both tissue and temperature. Contrasts were constructed as described in the edgeR manual. Gene Ontology [33] and Reactome [34] enrichments were found using the gseGO from the clusterProfiler [35] package, and gsePathway from the ReactomePA [36] package. Only gene ontologies with between 10 and 500 genes were investigated. Genes were marked as potentially secreted if they were present in one or more of the HPA, SignalP, Phobius & SPOCTOPUS list from the Human Protein Atlas [37]. Figures were generated in R version 4.4.0 using ggplot 2 version 3.5.1.

### 2.6 RNA extraction, cDNA synthesis and quantitative PCR

RNA extraction from cells *in vitro* was done using RNeasy Kit following the manufacturer's guidelines. One μg of RNA was converted to cDNA employing iScript cDNA synthesis kit (BioRad, Cat. 1708890) according to the instructions of the manufacturer. Real-Time PCR (RT-qPCR) was performed using Brilliant III Ultra Fast SYBR Green qPCR Master Mix (AH Diagnostics, Cat. 600883) in a CFX384 Real-Time Bio-Rad System. Raw CT data were normalized to the *Cyclophilin* reference gene for adipose tissue fractions and *Tbp* reference gene for cells following ΔΔ-Ct calculation. All primers are listed in Table S1.

### 2.7 CXCL12 ELISA

For secreted CXCL12 quantification, BAT explants were incubated in DMEM high glucose (Gibco, Cat. 11965126) supplemented with 0.1% fatty acid-free BSA and 1% penicillin/streptomycin (P/S) for 2 h at 37 °C. BAT explant media was then collected, and cell debris was removed by centrifugation at 1000*g* for 5 min at room temperature (RT). 100 μl of explant media was added in duplicates onto the ELISA plate. For plasma samples, blood was collected from the tail vein into ethylenediaminetetraacetic acid (EDTA)-coated tubes and centrifugated at 1000 g for 5 min at 4 °C. 100 μl of plasma was added in duplicates onto the ELISA plate. CXCL12 ELISA assays (R&D systems, Cat. DY460) were performed according to the manufacturer's instructions.

### 2.8 RNAscope in situ hybridization and immunohistochemistry

BAT samples were harvested from mice following 3, 7 and 21 days of thermoneutrality or cold exposure and fixed in 4% formaldehyde (PFA) (Sigma Aldrich, Cat. 818708) overnight. The tissue was dehydrated through absolute ethanol (VWR, Cat. 20821) and xylene (VWR, Cat. 28976.294) and embedded in paraffin blocks. Three μm tissue sections were deparaffinized in xylene (2 × 5 min) and absolute ethanol (2 × 2 min) and the RNAscope® Multiplex Fluorescent V2 Assay was performed according to the manufacturer's instructions (Advanced Cell Diagnostics, Cat. 323100) with a HybEZ™ II Hybridization System (Advanced Cell Diagnostics). Probes used were Mm-Cxcl12 (Advanced Cell Diagnostics, Cat. 422711-C2), Mm-Cxcr4 (Advanced Cell Diagnostics, Cat. 425901), Mm-Ackr3-C3 (Advanced Cell Diagnostics, Cat. 482561-C3), Mm-Pdgfra-C2 (Advanced Cell Diagnostics, Cat. 480661-C2), Mm-Itgax-C2 (Advanced Cell Diagnostics Cat. 311501-C2), Mm-Cd8a-C3 (Advanced Cell Diagnostics Cat. 401681-C3), Mm-Cd3e-C3 (Advanced Cell Diagnostics Cat. 314721-C3) probes, positive control probe Mm-Ppib (Advanced Cell Diagnostics, Cat. 313911-C2), or negative control probe DapB (Advanced Cell Diagnostics, Cat. 310043). The Mm-Ppib probe for mouse housekeeping gene *Ppib* was used as a control to ensure RNA quality. Opal reagents (Akoya, Cat. OP-001003, OP-001006) was used for signal visualization before the slides were blocked in 5% donkey serum (Sigma, Cat. D9663-10 ML) for 1 h at RT and incubated with goat Perilipin 1 (1:100, Abcam, Cat. Ab61682), rabbit alpha smooth muscle Actin (1:1000, Abcam, Cat. Ab124964), or rabbit CD68/SR-D1 (1:200, R&D Systems, Cat. MAB101141) antibodies at 4 °C overnight. The following day, slides were washed in PBS + 0.1% Triton X-100 and incubated in donkey anti-rabbit or donkey anti-goat secondary antibody (1:800, ThermoFisher Scientific, Cat. A21206, A-31573, A11055) for 1 h at RT. After washing, the slides were mounted with ProLong Gold antifade reagent with DAPI (Invitrogen, Cat. P36931) and visualized using a Zeiss Axio Observer microscope. The number of *Cxcl12*-positive cells, including both adipocytes and non-adipocytes, was evaluated using QuPath software.

### 2.9 WT-1 mouse cell line culture and differentiation

Brown WT-1 preadipocytes (kindly provided by C. R. Kahn) [38] were propagated and differentiated as previously described [27]. Briefly, preadipocytes were propagated in DMEM high glucose (Gibco, CAT. 11965126) with 10% fetal bovine serum (FBS) (Sigma, Cat. F7524) and 1% penicillin-streptomycin (P/S) (Gibco, Cat. 15070-063) (complete medium) in humidified incubators at 37 °C and 5% CO_2_ until they reached 80% confluency (d. −3). Cells were then incubated in complete medium supplemented with 20 nM insulin (Sigma, Cat. 192728) and 1 nM triiodothyronine (T_3_) (Sigma, Cat. T2877) (induction medium). At day 0, cells were incubated in induction medium supplemented with 0.125 mM indomethacin (Sigma Aldrich, Cat. 4902), 0.5 μM dexamethasone (Sigma Aldrich, Cat. 4902) and 0.5 mM 3-Isobutyl-1-methylxanthine (IBMX) (Sigma Aldrich, Cat. 15879) (differentiation medium). Two days after induction of differentiation (d. 2), the differentiation medium was replaced with induction medium, and was refreshed again 2 days later (d. 4). At day 6 after induction of differentiation (d. 6) media was refreshed to complete medium.

### 2.10 siRNA-mediated loss-of-function in WT-1 cells

siRNA knockdown was performed by reverse transfection as previously described [39]. Briefly, 5 μl/ml lipofectamine RNAiMAX (ThermoFisher Scientific, Cat.13778100) and 50 nM ON-Targetplus SMARTpool mouse Cxcl12 siRNAs (Dharmacon, Cat. L-044397-00) or Non-Targeting Control Pool (Dharmacon, Cat. D-001810-10-05) were used. For loss-of-function (LOF) experiments in mature adipocytes, cells were transfected 4 days after induction (d.4), refreshed on d.6 with complete medium and harvested on d.7 for analysis.

### 2.11 Generation and culture of WT-1-SAM stable cell line

The WT-1-SAM cell line was generated by stably expressing the CRISPRa-SAM components in brown WT-1 cells as previously described [40]. Briefly, HEK293-FT cells were transfected to produce lentiviruses expressing dCas9-VP64 and MS2-P65-HSF1. The virus containing supernatant was harvested 72 h post-transfection and added onto WT-1 cells. The successfully transduced cells were selected with 2.5 μg/ml blasticidin and 200 μg/ml hygromycin, and the expression of the CRISPRa-SAM components was validated by RT-qPCR. WT-1-SAM cells were cultured and differentiated as described for WT-1 cells.

### 2.12 CRISPRa-SAM mediated gain-of-function in WT-1-SAM cells

sgRNA design and cloning into sgRNA (MS2) cloning backbone vector (Addgene, #61424) for gain-of-function in WT-1-SAM cells was performed as previously described [40]. For *Cxcl12* over-expression experiments, WT-1-SAM cells were reverse-transfected on d.4 after induction of differentiation with 500 ng of *Cxcl12* sgRNA plasmid DNA (sgRNA sequence: TCTCTGACGCGCATCCCCTG) using TransIT-X2 (Mirus Bio, Cat. MIR6000). Media was refreshed on d.6 with complete medium and the transfected cells were harvested on d.7 for analysis.

### 2.13 J774A.1 macrophage cell line

J774A.1 macrophages in naïve state were purchased from ATCC (Cat. TIB-67) and propagated in DMEM high glucose supplemented with 10% FBS and 1% P/S until 85% confluency in humidified incubators at 37 °C and 5% CO_2_.

### 2.14 SH-SY5Y neuronal cell line culture and differentiation

SH-SY5Y neuroblastoma cells were purchased from ATTC (Cat. CRL-2266) and propagated in Minimum Essential Medium (MEM) (Gibco, Cat. 42360-024) supplemented with 15% FBS, 1% P/S and 1% non-essential amino acids (NEAA) (ThermoFisher Scientific, Cat. 11140050). Prior to experiments, cells were maintained until 85% confluency in a 5% CO_2_ humidified incubator at 37 °C. For cell differentiation, cultured cells were plated at a cell density of 1.6 × 10^5^ on 24-well plates (Corning, Cat. 3524). 24 h after, the medium was replaced with MEM cell culture medium supplemented with 1% FBS, 1% P/S and 1% NEAA (differentiation medium). Differentiation medium was refreshed every 2 days for a total of 6 days. Cells were observed during the experiment under a brightfield microscope to assess neuronal morphology and viability.

### 2.15 Free glycerol assay

7 days after induction of differentiation, cell medium was refreshed and supplemented with 1 μM norepinephrine (NE) (Sigma Aldrich, Cat. A9512) for 6 h. Media were then harvested and free glycerol levels were measured with a Free Glycerol Reagent (Sigma Aldrich, Cat. F6428) and Glycerol Standard Solution (Sigma Aldrich, Cat. G7793) according to the instructions of the manufacturer.

### 2.16 Mitochondrial respiration

Real-time measurements of oxygen consumption rates (OCR) and extracellular acidification rate (ECAR) were performed using a Seahorse XF96 Extracellular Flux Analyzer (Agilent Technologies). The cell culture medium was changed 1 h before the first measurement to XF assay medium DMEM (Agilent Technologies, Cat. 103575-100) supplemented with 5 mM Glucose (Agilent Technologies, Cat. 103577-100) and 2 mM l-glutamine (Agilent Technologies, Cat. 103579-100). For the uncoupling assay, OCR was measured under basal conditions and after injection of oligomycin (5 μM) (Agilent Technologies) and NE (1 μM). Area under the curve (AUC) was calculated from the time of the NE injection. For the Mito Stress Test, OCR was measured under basal conditions and following injection of oligomycin (5 μM), FCCP (0.5 μM) and Rotenone (1 μM) combined with Antimycin A (1 μM) (all from Agilent Technologies). Calculations were performed by the Seahorse report generator.

### 2.17 Immunoblotting

Cell protein lysates were prepared as previously described [27]. Briefly, cells were lysed in RIPA lysis buffer (Pierce, Cat. 89901) supplemented with protease inhibitors (Sigma, Cat. S8820) and phosphatase inhibitors (1 mM Na_3_VO_4_, 10 mM β-Glycin, 10 mM NaF and 10 mM Na_4_P_2_O_7_). Cell lysates were centrifuged at 15000 g for 10 min at 4 °C and the supernatant was collected. Samples were subjected to 95 °C reduction for 5 min in 20% sample buffer (0.35 M Tris–HCl; 10% SDS; 30% glycerol; 603 mM DTT and 150 μM bromophenol blue). 25 μg of protein was used per lane and loaded onto 4-15% Criterion TGX Precast midi gels (BioRad, Cat. 5671084) and run in MES-SDS running buffer (ThermoFisher Scientific, Cat. NP0002) for 20 min at 60 V and then changed to 120 V for 1 h and 20 min. Gels were transferred onto Immobilon PVDF membranes (Milipore, Cat. IPVH00010) and blocked with 5% skimmed milk (Milipore, Cat. 70166) in TBS-Tween (0.1%) for 1 h at RT. Membranes were incubated in primary antibodies overnight at 4 °C followed by incubation with anti-rabbit HRP-conjugated secondary antibody (1:4000; Bio-Rad, Cat. 1706515) for 1 h at RT. The membranes were developed with Immobilon Forte Western HRP substrate (Millipore, Cat. WBLUFD100) employing a ChemiDoc XRS + Molecular Imager (Bio-Rad, Cat. 1708265). Primary antibodies and dilutions are listed in Table S2.

### 2.18 Neurite outgrowth and immunocytochemistry

SH-SY5Y cells were differentiated as described above supplemented with 10 μM retinoic acid (Sigma Aldrich, Cat. R2625) or 0.01, 0.1 and 1 μM recombinant human CXCL12 (R&D Systems, Cat. 350-NS-050). SH-SY5Y cells were fixed in 4% PFA for 15 min before being incubated with permeabilization solution (0.5% Triton X-100, Sigma Aldrich, Cat. 93443) for 15 min. Nonspecific antibody binding was blocked with 3% BSA for 1 h at RT. Cells were stained with primary antibody against B-tubulin III (1:1000, Abcam, Cat. Ab 18207), and Alexa fluor plus 488 anti-rabbit secondary antibody (1:1000, Thermofisher, Cat. A48254) for 1 h at RT. Prior to imaging, cell nuclei were stained with NucBlue Fixed Cell ReadyProbes Reagent (DAPI) (ThermoFisher Scientific, Cat. R37606). Neurite outgrowth events in individual wells were measured as tracing overlays quantifying total or mean neuronal outgrowth, processes, branches, and cell viability from images acquired with the ImageXpress Pico system (Molecular Devices) and analyzed by the MetaXpress software. The total outgrowth of the neurons was normalized to total cell count.

### 2.19 Chemotaxis assays

For chemotaxis assays with recombinant CXCL12, 1 μM recombinant mouse CXCL12 (R&D systems, Cat. 460-SD-010/CF) was diluted in starvation media (DMEM high glucose supplemented with 0.1% fatty acid-free BSA and 1% P/S) and placed on the bottom of the well-plate. Previously equilibrated cell culture inserts (Corning, Cat. CLS3422) were placed in the cell-containing wells with 250,000 cells/ml of macrophages and incubated at 37 °C for 3 h. For co-culture experiments, WT-1 or WT-1-SAM cells were grown, differentiated, and transfected as described above. On d.6 after induction of differentiation, media was refreshed to starvation media. The next day, previously equilibrated cell culture inserts were placed in cell-containing wells with 250,000 cells/ml of macrophages and incubated at 37 °C for 6 h. After incubation, cell media from the inserts was removed and the top was washed using a cotton swab. The cells were fixed in ice-cold methanol (Sigma Aldrich, Cat. 322415) for 10 min at RT prior to staining with DAPI (BD Pharmingen, Cat. 564907) or propidium iodide (Sigma Aldrich, Cat. P4864) solution (1:1000; 0.3% Triton-X100) for 10 min at RT. The inserts were washed in distilled water and air dried. The migrated cells were observed under a Zeiss AxioZoom microscope and quantified employing an intellesis trainable segmentation with Zeiss Blue Zen software.

### 2.20 Statistical analysis

All experiments were performed as three or more independent biological replicates. Data are represented as mean ± SEM with individual data points. All statistical tests were performed using GraphPad Prism 9 software (GraphPad). For comparisons between two groups, unpaired or paired, two-tailed Student's t-test was performed, depending on the experiment. Multiple comparison tests were performed using one- or two-way ANOVA, followed by post-hoc tests as recommended by GraphPad PRISM 9 software (Šídák's, Dunnett's or Tukey's multiple comparison tests were applied after a two-way ANOVA, and the Dunnett's post-hoc test was used after one-way ANOVA). Values below 0.05 (p < 0.05) were considered significant. (∗) p < 0.05; (∗∗) p < 0.01; (∗∗∗) p < 0.001; (∗∗∗∗) p < 0.0001.

### 2.21 Data availability

The RNA-sequencing data are publicly available on GEO database with the accession number: GSE242711.

## 3 Results

### 3.1 Depot-specific transcriptomic signatures of mature adipocytes in response to long-term cold acclimation

**Figure 1:**
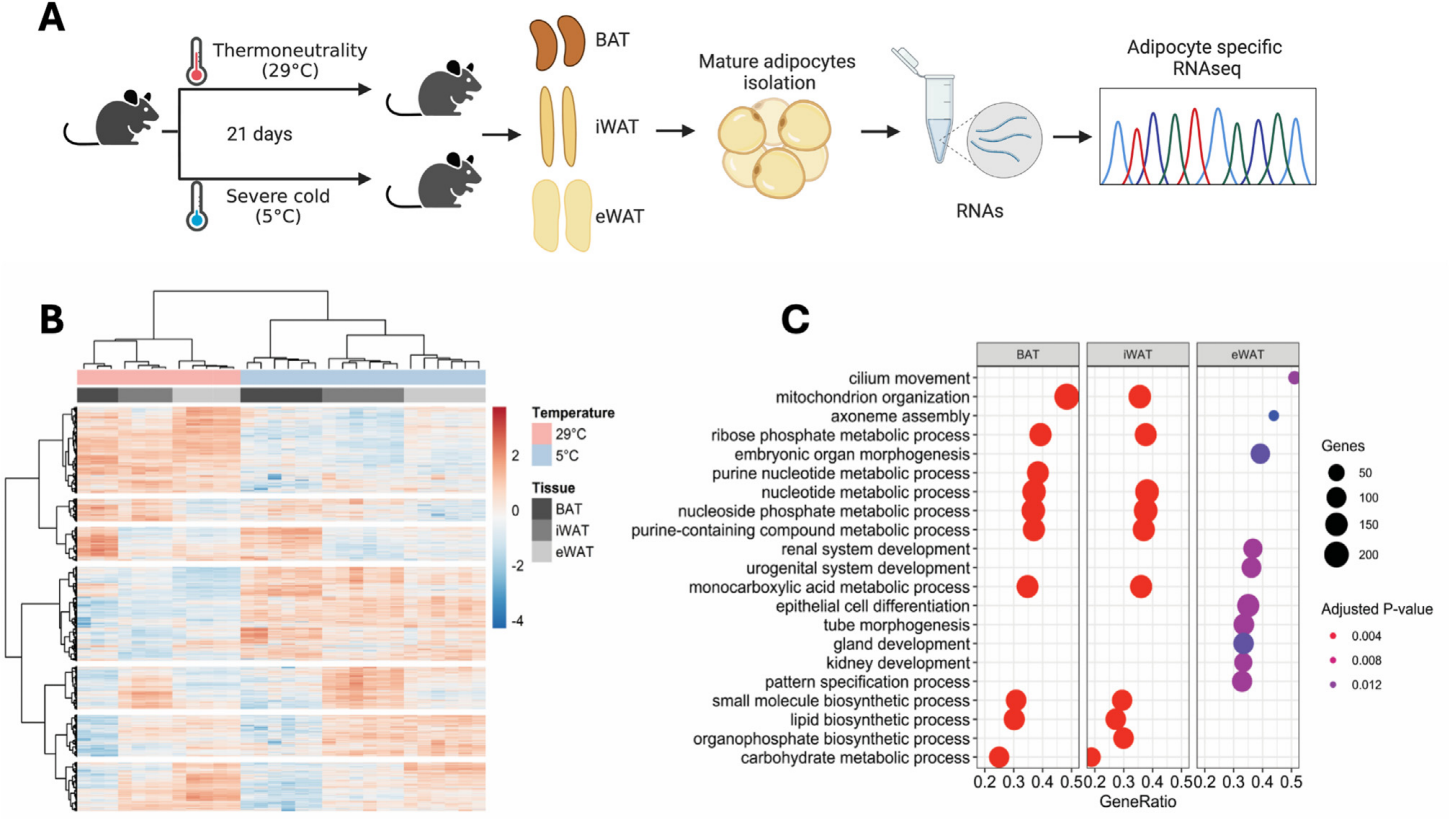
**Depot-specific transcriptomic signatures of mature adipocytes in response to long-term cold acclimation. A.** Study design: environmental temperature exposure and transcriptomic profiling of mature adipocyte fractions from brown (BAT), inguinal (iWAT) and epididymal adipose tissue (eWAT). **B.** Heatmap illustrating depot-specific adipocyte gene clusters at thermoneutrality (TN, 29 °C) or cold (CE, 5 °C). TN group n = 3–5; CE group n = 6. **C.** Gene Ontology analysis of significantly regulated genes of adipocytes from BAT, iWAT and eWAT.

To obtain an adipocyte-selective and brown adipocyte-enriched library of transcripts, we performed deep RNA sequencing in mature adipocytes isolated from murine brown (BAT), inguinal white (iWAT) and epididymal white (eWAT) adipose tissues. Mice were housed at thermoneutrality (29 °C) for 21 days, and either kept at thermoneutrality or transferred to cold (5 °C) for 21 days to determine the transcriptomic changes to physiological cold adaptation (Figure 1A). We identified 7 major clusters of genes with distinct transcript identities, 5 of which showed depot-specific profiles at thermonutrality and after chronic epoxsure to cold (Figure 1B). As expected, there was a clear segregation of adipocytes from the 3 fat depots, regardless of the environmental temperature, based on principal component analysis (PCA) (Figure S1A), yet adipocytes from BAT and iWAT, a depot capable of browning, had the most similar transcriptomic profiles upon chronic cold challenge (Figure S1B). Overall gene ontology (GO) analysis confirmed major differences between brown/beige and white adipocytes (Figure 1C), where *mitochondrial organization*, *substrate biosynthesis and breakdown* (lipid, carbohydrate) as well as *nucleotide metabolic processes* were the most over-represented pathways in adipocytes from BAT and iWAT, while *tissue morphogenesis* and *epithelial cell differentiation* were more prominent in eWAT (Figure 1C).

Of all detected transcripts, 858 genes were altered with chronic cold exposure in BAT adipocytes, 70 in iWAT and 13 in eWAT adipocytes (FDR < 0.05; Figure S1C; Table S3). Of all cold-sensitive transcripts in BAT adipocytes, many encoded mitochondrial proteins and classic thermogenic markers such as very long-chain fatty acid elongation protein, *Elovl3*, *Elovl6*, *Ucp1*, *Cidea* and multiple enzymes involved in Acyl-CoA synthesis (*Acsl5*, *Acss2*), esterification (*Acot11*) and carboxylation (*Acaca*, *Acacb*). Similarly, adipocytes isolated from iWAT exhibited drastic elevations in *Ucp1*, *Elovl3*, *Cidea*, *Elovl6*, *Acot11*, *Cox8b* and *Dio2* in the cold-exposed group compared to thermoneutrality, illustrating the presence of fully reprogrammed beige/brite adipocytes that are equipped to handle fatty acid oxidation after chronic acclimation to cold. In contrast, among the 13 genes altered with cold in visceral adipocytes were members of extracellular matrix (ECM) remodeling including *Mmp12* and *Spon 1*, while thermogenesis-related genes were neither expressed or changed after chronic cooling. Collectively, these data confirm depot-dependent responses to environmental cold and provide important insights into adipocyte-specific transcriptional signatures; offering higher resolution compared to standard bulk tissue analysis, where signal from mature adipocytes is diluted or lost due to the highly heterogenous composition of adipose tissues.

### 3.2 Cold acclimation reprograms the brown adipocyte secretome

**Figure 2:**
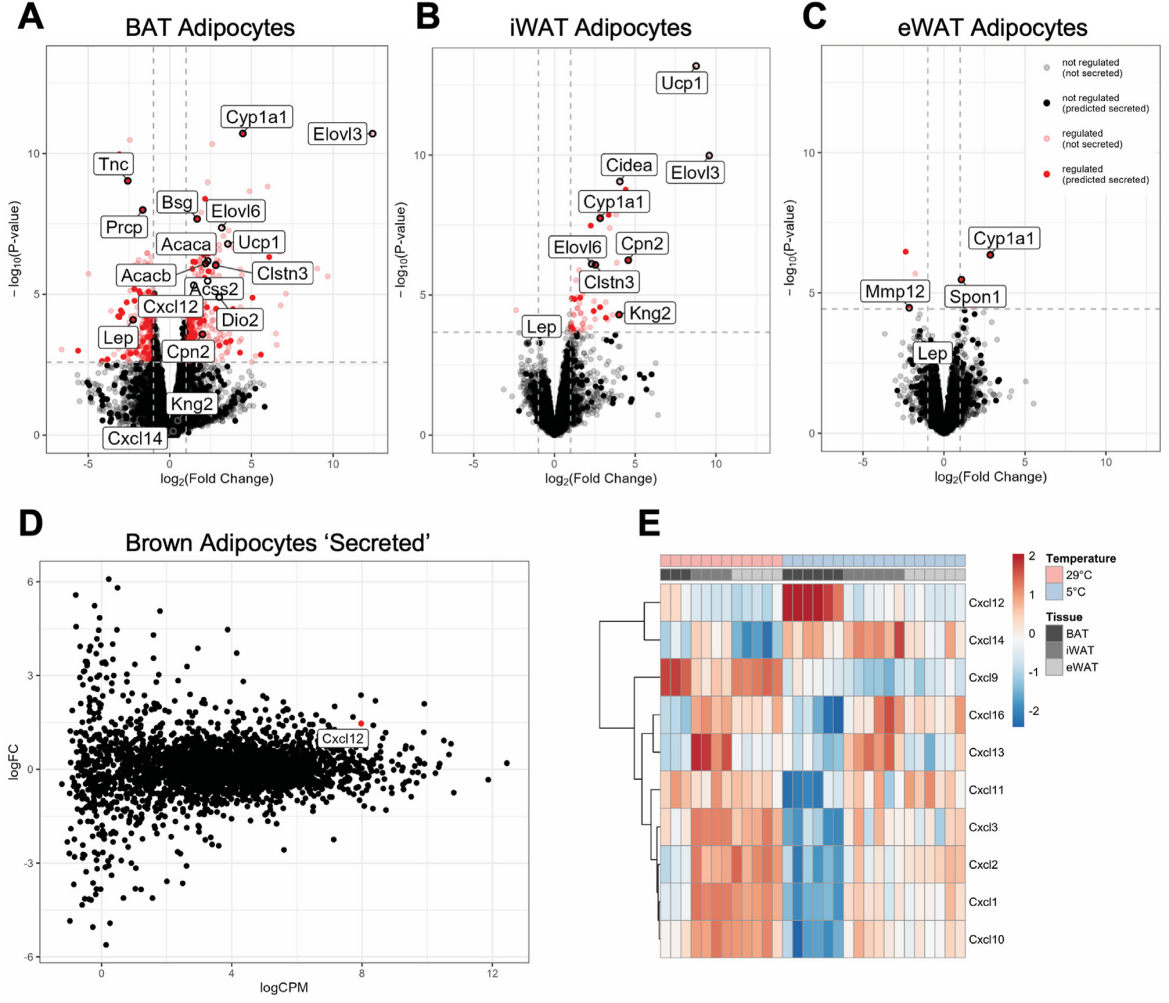
**Cold acclimation reprograms mature adipocyte secretome. A-C.** Differentially expressed genes (5 °C/29 °C), including not secreted (pink) and predicted to be secreted (red) transcripts in BAT-derived brown adipocytes (**A**), inguinal adipocytes (**B**) and epididymal adipocytes (**C**). **D.** MA plot depicting the magnitude of change in gene expression between thermoneutrality and chronic cold exposure according to the mean gene expression for all predicted to be secreted BAT-derived factors. **E.** Heatmap illustrating CXC family gene expression across adipocytes from BAT, iWAT and eWAT depots of mice exposed to thermoneutrality (TN, 29 °C) and cold conditions (CE, 5 °C). TN group n = 3–5; CE group n = 6.

To identify secreted factors from fully activated brown adipocytes that may contribute to tissue homeostasis during increased energy demand and/or whole-body energy expenditure, we next sought out to look specifically for genes that encode secreted proteins and survey BAT-enhanced mediators. Of 16460 transcripts in BAT adipocytes, 2952 were identified as coding for secreted proteins upon analysis by classical prediction methods (HPA, SignalP, Phobius, Spoctopus), including ER-resident proteins, fully transmembrane or membrane-docked proteins and *bona fide* signaling mediators that locate to extracellular space. 210 of these transcripts were differentially expressed between thermoneutral and cold-exposed BAT-adipocytes (FDR < 0.05; Figure 2A), while 16 of 70 cold-regulated iWAT transcripts were predicted to be secreted (FDR < 0.05; Figures 2B and 4 of 13 in eWAT (FDR < 0.05; Figure 2C). Interestingly, two gene transcripts were significantly induced by cold in all fat depots (Figure S1D) - cytochrome P450 family 1, subfamily A, polypeptide 1 (*Cyp1a1*) and Serine/Cysteine peptidase inhibitor member 3 K (*Serpina3k*). Notably, however *Cyp1a1* was most abundant in brown adipocytes, while *Serpina3k* remained the most enriched in adipocytes from eWAT (for absolute quantification of all transcripts across depots, see Table S3). Among the commonly regulated ‘secretome’ between brown and beige adipocytes included Calsyntenin 3 (*Clstn3*), a gene involved in innervation and multilocularity of thermogenic fat depots [25,41,42], Carboxypeptidase N Subunit 2 (*Cpn2*), and Kininogen 2 (*Kng2*), a component of the kallikrein-kinin system regulating BAT thermogenesis [43]. We next prioritized transcripts that were upregulated by cold, with a clear enrichment in BAT over iWAT and eWAT (Table S3), with eWAT being the least thermogenic depot hence providing the most striking contrast with BAT, and with none or weak transmembrane association, as determined using the TMHMMv2.0 software [44]. We found C-X-C motif chemokine ligand 12 (CXCL12) among the most abundant, top BAT-enhanced and cold-induced secreted factor encoding transcripts (Figure 2D), whose levels tripled after chronic acclimation to cold (FDR = 0.007). Interestingly, the expression profile of CXC chemokines in discrete fat depots was dramatically altered upon cold adaptation, with *Cxcl12* clearly becoming the most abundant one in adipocytes from BAT (Figure 2E). We thus further pursued CXCL12 as a uniquely regulated chemokine in BAT and plausible brown adipocyte-enriched secreted factor with unknown function in this depot.

### 3.3 Cold exposure promotes *Cxcl12* expression and CXCL12 secretion in brown adipocytes

**Figure 3:**
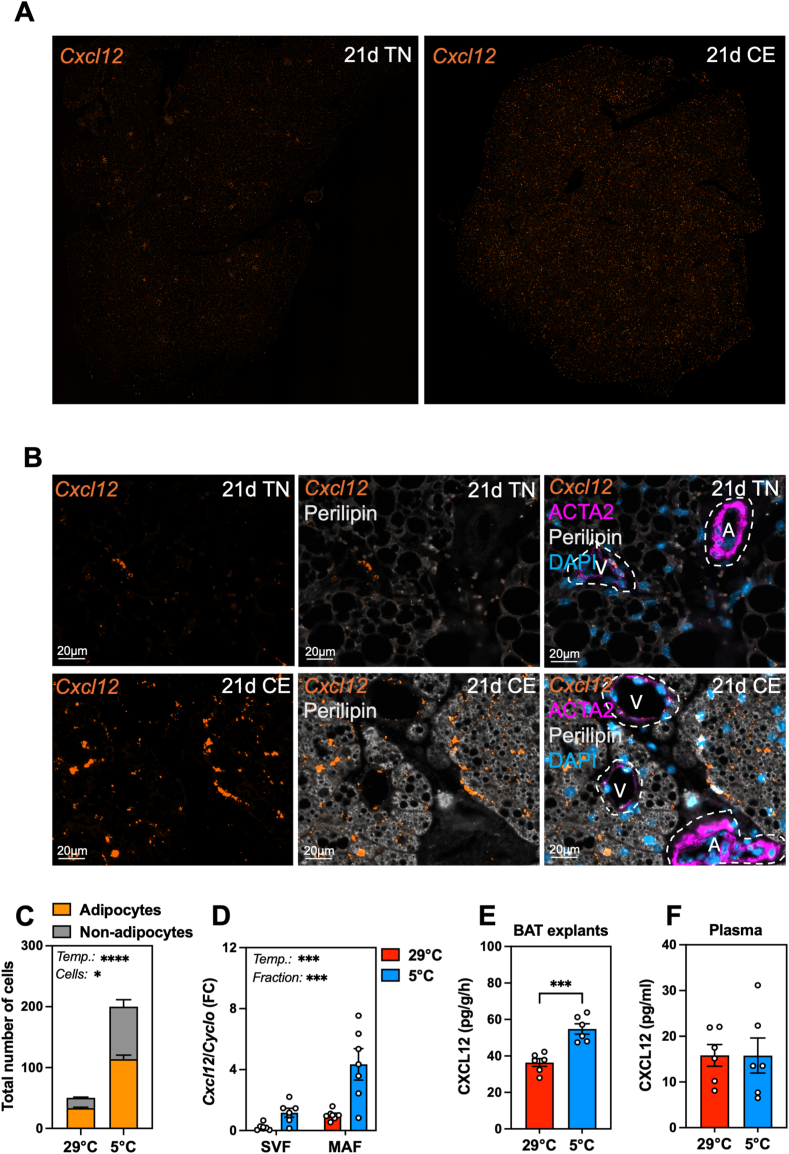
**Cold exposure promotes *Cxcl12* expression and CXCL12 secretion in brown adipocytes. A.** RNA-scope fluorescence in situ hybridization (FISH) for *Cxcl12* (orange) in BAT following 21D thermoneutrality housing (21  d TN) or cold exposure (21 d CE) (representative image) (n = 3). **B.** FISH for *Cxcl12* (orange) with immuno-histochemistry for perilipin (white) and alpha-smooth muscle actin (ACTA2, magenta), and DAPI staining (blue) in BAT, following 21 days exposure to cold (5 °C) (representative image) (n = 3). Areas with low ACTA2 staining (representative of veins, labeled as V) and high ACTA2 staining (representative of arteries, labeled as A) are indicated with dashed lines. **C.** Estimation of *Cxcl12*-positive adipocytes and non-adipocyte cells per field of view at thermoneutrality and upon 21 d cold exposure. For statistical analysis, samples from three animals were used, with three images per sample acquired at 20 × magnification. **D.** mRNA expression of *Cxcl12* in the stromal vascular fraction (SVF) and mature adipocyte fraction (MAF) isolated from BAT of mice exposed to thermoneutrality (29 °C) and cold conditions (5 °C) (n = 5–6). **E.** Secretion rate of CXCL12 by BAT explants after 21 d at thermoneutrality (29 °C) or cold exposure (5 °C) (n = 6). **F.** Secreted CXCL12 levels in plasma after 21 d at thermoneutrality (29 °C) or cold (5 °C) (n = 6). Statistical significance was performed using t-test (unpaired, two-tailed) (p < 0.05) or 2-way ANOVA (α < 0.05). (∗) p < 0.05; (∗∗) p < 0.01; (∗∗∗) p < 0.001.

To explore the cold regulation of *Cxcl12* in BAT, we performed RNAscope fluorescence in situ hybridization (FISH), which revealed a progressive increase in *Cxcl12* transcript abundance in BAT over 3, 7 and 21 days of cold acclimation compared to thermoneutral conditions (Figure 3A; Figure S2A). To obtain a better insight into the subpopulations expressing *Cxcl12*, we used immunohistochemistry co-staining for Perilipin (adipocytes) and ACTA2 (smooth muscle cells). These data illustrated that increased *Cxcl12* expression occurred in brown adipocytes during prolonged exposure to cold, as well as in other non-adipocyte cells (Figure 3B, C). Interestingly, divergent *Cxcl12* expression patterns were observed in ACTA2^low^ (veins) and ACTA2^high^ (arteries) cells, with ACTA2^low^ smooth muscle cells expressing *Cxcl12* both under thermoneutrality and cold conditions (Figure 3B). Comparative analysis of *Cxcl12* regulation in mature adipocyte fraction (MAF) and stromal vascular fraction (SVF) from BAT indicated that *Cxcl12* expression was enhanced upon cold exposure in both fractions, and the highest *Cxcl1*2 mRNA levels were found in the MAF alongside *Adipoq* (Figure 3D; Figure S2B), suggesting mature brown adipocytes to be a major, although not unique, source of CXCL12 in cold-activated BAT. To investigate whether cold exposure directly stimulates CXCL12 secretion from BAT to target distal tissues, we quantified CXCL12 levels in BAT explant media and plasma of mice exposed to cold for 3, 7 and 21 days. CXCL12 secretion from BAT was found to be increased by cold, with the highest secretion rate reached upon 21 days of cold acclimation, as compared to thermoneutral conditions (Figure 3E; Figure S2C). By contrast, CXCL12 levels in plasma did not differ among conditions (Figure 3F), suggesting a local action of this chemokine in BAT. Collectively, these data indicate an induction of CXCL12 in brown mature adipocytes upon cold activation, which can be secreted into the extracellular milieu, potentially acting on neighboring cells.

### 3.4 Mature brown adipocytes require CXCL12 for optimal thermogenic response

**Figure 4:**
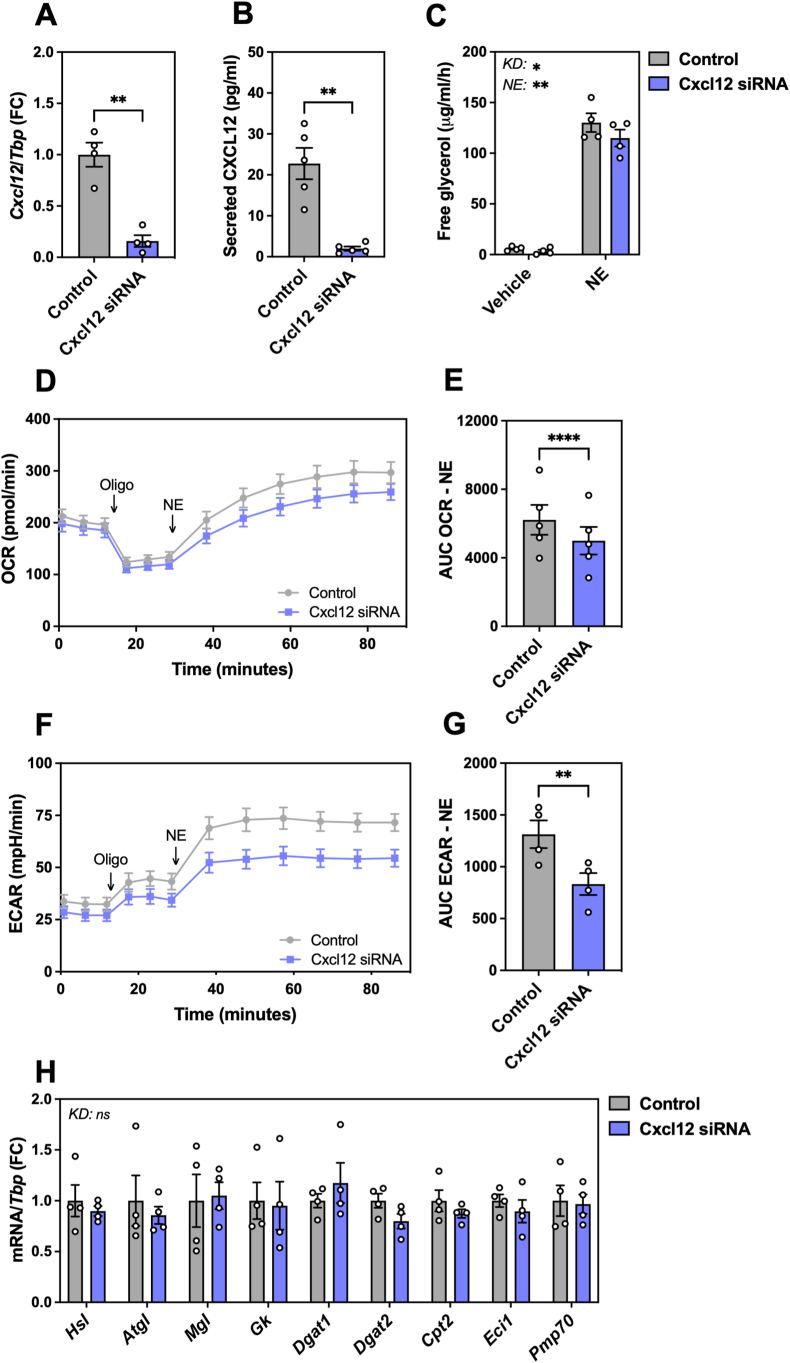
**Mature brown adipocytes require CXCL12 for optimal thermogenic response**. **A-B.** Knockdown of *Cxcl12* in murine mature brown adipocytes reduces *Cxcl12* expression **(A)** (n = 4) and CXCL12 secretion **(B)** (n = 5). **C.** Free glycerol content in the media of control and *Cxcl12* KD brown adipocytes with or without (Vehicle) NE stimulation (n = 4). **D-E.** Mitochondrial respiration (OCR: oxygen consumption rate) in response to NE in control and *Cxcl12* KD murine mature brown adipocytes, **(D)** representative experiment and **(E)** quantification (n = 5). **F-G.** Extracellular acidification rate (ECAR) in response to NE in control and *Cxcl12* KD murine mature brown adipocytes, **(F)** representative experiment and **(G)** quantification (n = 4). **H**. Relative expression (RT-qPCR) of selected genes mediating lipolysis (*Hsl*, *Atgl*, *Mgl*), triglyceride esterification (*Gk*, *Dgat1*, *Dgat2*), and fatty acid oxidation (*Cpt2*, *Eci1*, *Pmp70*), normalized to *Tbp*. Statistical significance was performed using a t-test (paired, two-tailed) (p < 0.05) or a 2-way ANOVA (α < 0.05). (∗) p < 0.05; (∗∗) p < 0.01; (∗∗∗) p < 0.001; (∗∗∗∗) p < 0.0001.

**Figure 5:**
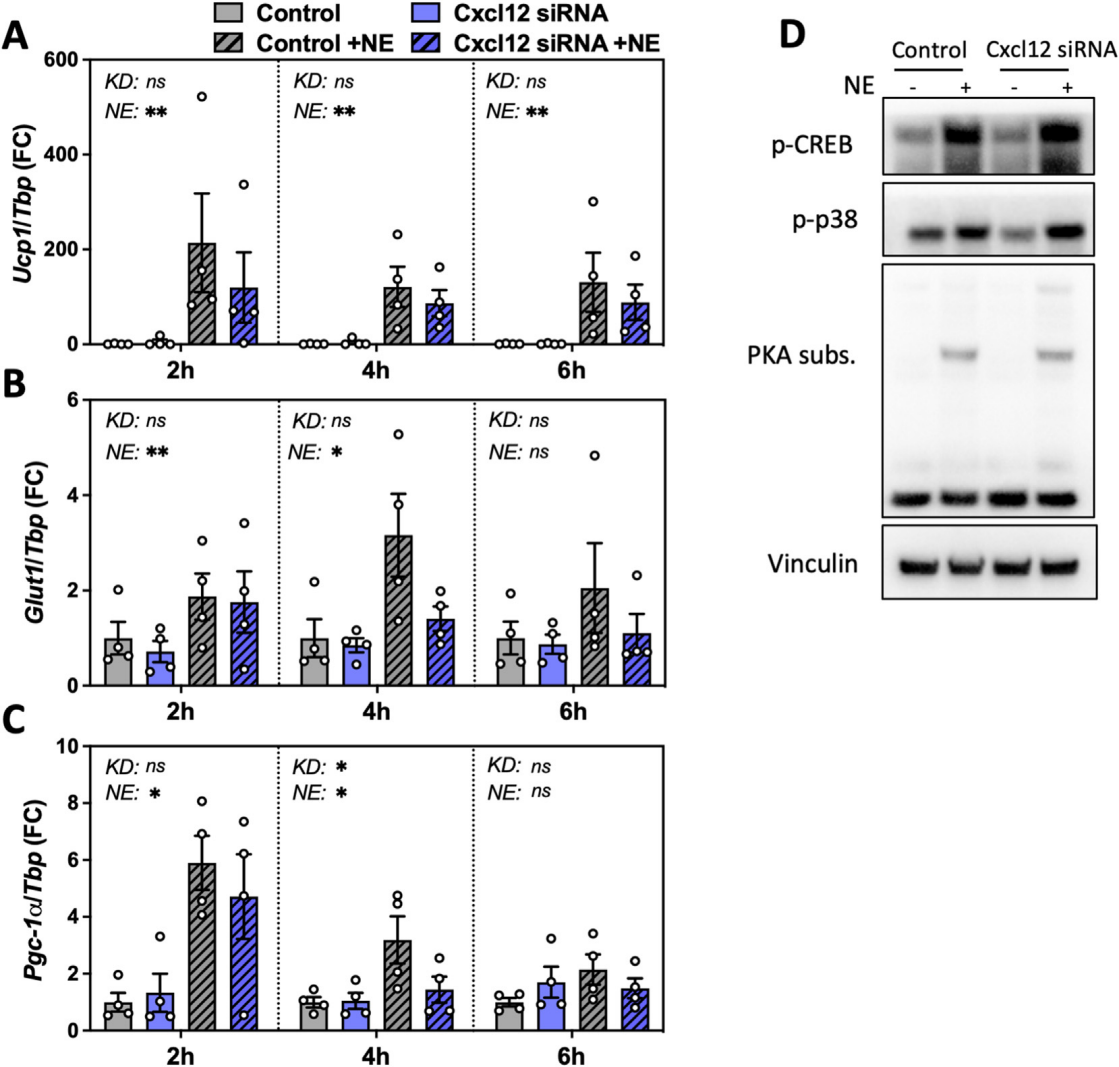
**Impact of reduced CXCL12 secretion on NE-induced thermogenic gene expression and the β-adrenergic signaling pathway in brown adipocytes. A-C.** mRNA expression of *Ucp1***(A)**, *Glut1***(B)** and *Pgc-1α***(C)** in controls and *Cxcl12* KD murine mature brown adipocytes with/without NE stimulation for 2, 4 and 6 h (n = 4). **D.** Immunoblotting of p-CREB, p-p38 and PKA substrates in controls and *Cxcl12* KD brown adipocytes after NE stimulation (representative experiment, n = 4). Statistical significance was performed using an 2-way ANOVA (α < 0.05). (∗) p < 0.05; (∗∗) p < 0.01; (∗∗∗) p < 0.001; (∗∗∗∗) p < 0.0001.

To determine the role of CXCL12 produced by brown adipocytes, we first investigated whether CXCL12 had any impact on brown adipocyte function *in vitro*. We tested the effect of elevated CXCL12 secretion using a gain-of-function model employing CRISPRa-mediated transactivation [40] to increase endogenous *Cxcl12* expression (Figure S3A) and CXCL12 secretion (Figure S3B). Boosting CXCL12 release did not alter norepinephrine (NE)-induced *Ucp1* mRNA expression (Figure S3C), glycerol release (Figure S3D) nor mitochondrial respiration (Figure S3E–H). On the other hand, *Cxcl12* silencing using siRNA transfection in mature brown adipocytes (Figure 4A) nearly abolished CXCL12 secretion into the extracellular medium (Figure 4B). Loss of *Cxcl12* function had no effect on the expression on key adipogenic marker genes in brown adipocytes, including *Pparg*, *Fabp4* and *Adipoq* (Figure S3I), nor on mitochondrial performance (basal respiration, ATP-linked respiration, maximal and reserve capacities; Figure S3J), indicating that the absence of CXCL12 did not impact brown adipocyte maturation. However, when brown adipocytes were stimulated with norepinephrine (NE), we observed a modest reduction in extracellular glycerol concentration (p < 0.05; Figure 4C), as well as decreased oxygen consumption and extracellular acidification rates (Figure 4D–G) in cells lacking *Cxcl12* as compared to controls, which are indicative of an altered thermogenic response. The expression of genes regulating lipolysis, esterification of triglycerides, fatty acid oxidation or involved with UCP1-independent energy dissipation remained unchanged under these conditions (Figure 4H; Figure S3K). These results suggest a cell-autonomous process involving endogenous CXCL12 secretion to support NE-mediated energy dissipation, possibly by a cross-talk with the β-adrenergic signaling pathway. To further investigate the interplay between CXCL12 and NE in brown adipocytes, we measured the differential expression of key thermogenic genes upon stimulation with NE in the presence or absence of CXCL12. As expected, *Ucp1*, *Glut1* and *Pgc-1α* mRNA levels were all upregulated by NE stimulation, in a sustained manner for *Ucp1*, while *Glut1* and *Pgc-1α* mRNA levels returned to basal after 6 h (Figure 5A–C). While no difference was observed in *Ucp1* induction in *Cxcl12* knockdown cells as compared to controls, a quicker return to basal levels occurred for *Pgc-1α* (p = 0.0101) in cells lacking *Cxcl12* while a similar trend was observed for *Glut1* (p = 0.1483), suggesting a stronger or faster attenuation of the β-adrenergic signaling in brown adipocytes when CXCL12 release is abrogated. However, examination of the phosphorylation events downstream of the β-adrenergic pathway by western blotting (p-CREB, p-p38 or p-PKA substrates) did not reveal any differences between control and *Cxcl12* knockdown cells (Figure 5D). This suggests that the functional cross-talk between CXCL12 and NE in brown adipocytes may occur beyond a direct modulation of the cAMP/PKA pathway controlled by β-adrenergic receptor stimulation. Collectively, our results indicate that CXCL12 is required for brown adipocyte optimal performance upon stimulation by NE.

### 3.5 Brown adipocytes communicate with neighboring cells via CXCL12

**Figure 6:**
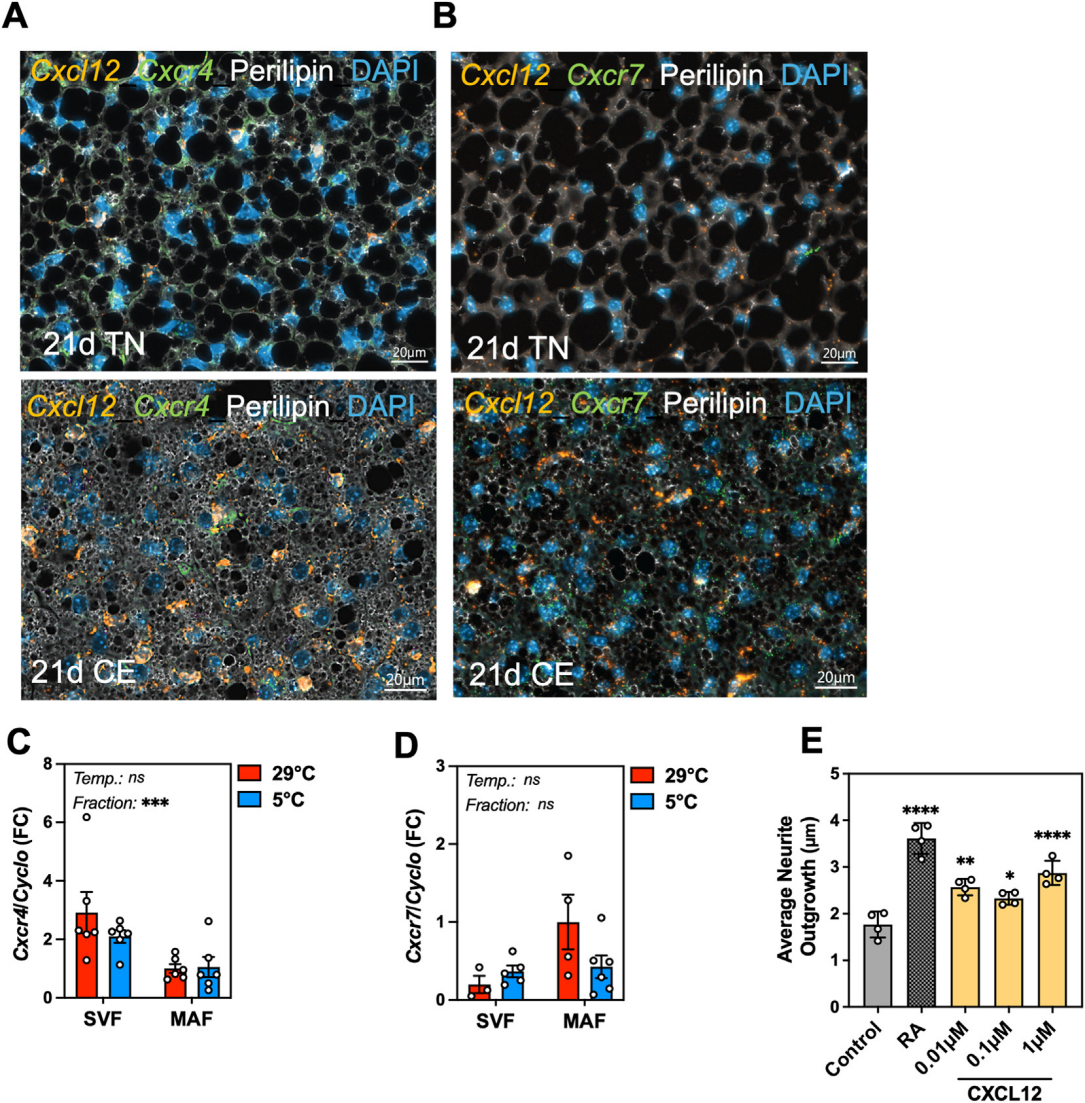
**Presence of CXCL12-sensitive cells in BAT. A-B.** FISH for (**A**) *Cxcr4* or (**B**) *Cxcr7* (green), *Cxcl12* (orange) and immunohistochemistry staining of perilipin (white) and DAPI (blue) in BAT at thermoneutrality **(left)** or following 21 days of cold exposure (5 °C) **(right)** (representative image, n = 3). **C-D.** Relative expression (RT-qPCR) of *Cxcr4* (**C**) and *Cxcr7* (**D**) in the stromal vascular fraction (SVF) and mature adipocyte fraction (MAF) isolated from BAT of mice exposed to thermoneutrality (29 °C) and cold conditions (5 °C) (n = 5–6). **E.** Average neurite outgrowth of SH-SY5Y neurons treated with retinoic acid (RA) or different concentrations of recombinant human CXCL12 (0.01, 0.1 and 1 μM CXCL12) (n = 4). Statistical significance was performed using one-way ANOVA or two-way ANOVA with Dunnett's multiple comparisons test. (∗) p < 0.05; (∗∗) p < 0.01; (∗∗∗) p < 0.001.

**Figure 7:**
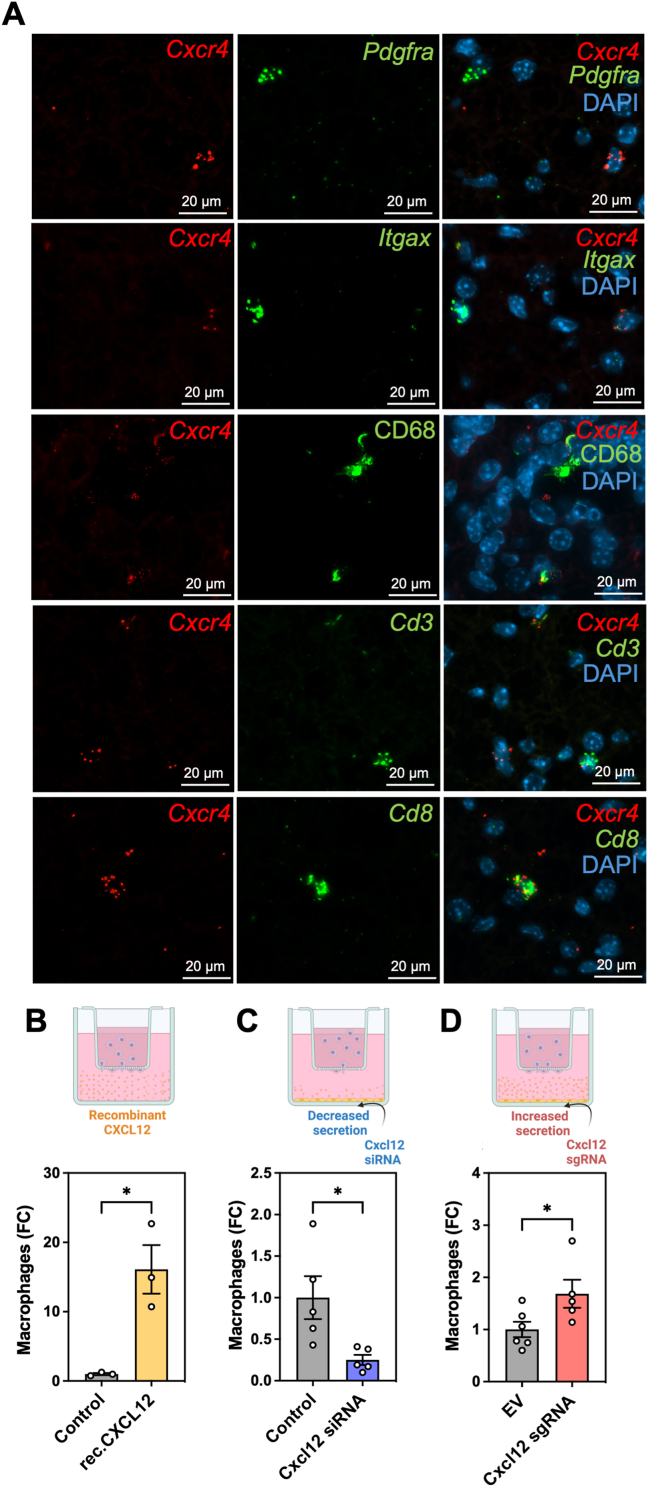
**CXCL12 induces chemotaxis of macrophages *in vitro*. A**. FISH for *Cxcr4* (pink) and for selected markers for multiple cell types including *Pdgfra* (adipocyte progenitors), *Itgax* (dendritic cells), *Cd3* and *Cd8* (T lymphocytes) (green) together with immuno-histochemistry for CD68 (macrophages, green), and DAPI (blue) in BAT following 7 days of cold exposure (5 °C) (representative image, n = 3). **B-D**. Macrophage migration (number of macrophages that migrated across the membrane) illustration and quantification (**B**) upon gradient generated by recombinant mouse CXCL12 (1 μM) (n = 3); (**C**) upon reduced CXCL12 secretion by mature brown adipocytes (n = 5); (**D**) upon increased CXCL12 secretion by mature brown adipocytes (n = 5). Statistical significance was performed using a t-test (unpaired, two-tailed); (∗) p < 0.05.

We hypothesized that CXCL12 may exert chemo-attracting properties on surrounding cells within BAT to facilitate tissue remodeling in adaptation to cold. To probe the potential paracrine function of CXCL12 in BAT, we first mapped the expression pattern of the two best characterized receptors for CXCL12, *Cxcr4* and *Cxcr7*, in the tissue, at various temperatures. Scattered patterns of *Cxcr4* and *Cxcr7* expressing cells were found surrounding *Cxcl12*-expressing brown adipocytes, both at thermoneutral conditions (Figure 6A left, 6 B left) and after cold exposure (Figure 6A right, 6 B right; Figure S4A, B), supporting a potential role for CXCL12 in orchestrating intercellular cross-talk in activated BAT to coordinate tissue homeostasis and composition. While *Cxcr4* was expressed in both SVF and MAF, its expression was higher in SVF and was not affected by ambient temperature (Figure 6C). *Cxcr7* was equally distributed among SVF and MAF and temperature had no effect on its expression levels (Figure 6D). Given the critical function of CXCL12 in regulating neurogenesis and immune cell migration in other organs [45], as well as the importance of neurogenesis and macrophage accrual in cold-induced BAT remodeling, we aimed to investigate potential cross-talk of CXCL12 with neurons and macrophages. To interrogate the ability of CXCL12 to communicate with sympathetic neurons, we tested the capacity of CXCL12 to control neurite branching in SH-SY5Y cells, a neuronal cell line capable of producing NE [46]. CXCL12 significantly potentiated neurite outgrowth as compared to the untreated control condition, almost reaching the maximum extension achieved with the retinoic acid positive control (Figure 6E; Figure S4C), providing direct evidence that SH-SY5Y cells were able to sense and respond to CXCL12. In addition, considering the putative engagement of *Cxcr4*-expressing cells such as immune cell types including macrophages and lymphocytes in BAT [47], we investigated *Cxcr4* spatial distribution in BAT and its co-expression with markers of selected cell-types. The presence of *Cxcr4* mRNA was found in CD68 (a general macrophage marker) positive cells, as well as in *Cd3* and *Cd8* (T lymphocyte markers)-expressing cells, but not in *Pdgfra* (adipocyte precursor) or *Itgax* (dendritic cells)-expressing cells, within BAT from cold-exposed mice (Figure 7A). This indicates that BAT resident macrophages, as well as some T lymphocytes, are equipped for CXCL12 sensing and suggests some paracrine communication during cold acclimation. To further characterize brown adipocyte-macrophage crosstalk, we performed a chemotaxis assay and measured macrophage migration. First, we confirmed the strong chemotactic capacity of recombinant CXCL12 on macrophages in a transwell cell migration assay (Figure 7B) (p = 0.0126). This occurred without any detectable change in maturation (Figure S4D, E). Next, we gauged CXCL12 contribution to the paracrine communication between brown adipocytes and macrophages using a co-culture system between macrophages and mature brown adipocytes in conditions of decreased (siRNA; Figure 4A, B) or induced (CRISPRa-SAM; Figure S3A, B) CXCL12 secretion. Decreased CXCL12 secretion from brown adipocytes significantly reduced macrophage migration (Figure 7C) (p = 0.0222), whereas the opposite outcome was observed upon heightened secretion of CXCL12 (Figure 7D) (p = 0.0442). Combined, these results support a role for CXCL12 as an active mediator of the communications emanated from brown adipocytes with neurons and macrophages *in vitro*.

## 4 Discussion

A deep understanding of the molecular and intercellular processes controling BAT homeostasis and remodeling are essential to exploit BAT activation as a therapeutic means to improve metabolic health. Some factors released from BAT were described to influence metabolism by tightly regulating the thermogenic depot microenvironment [48,49], highlighting the importance of outlining the BAT secretome. While the secretory profile of brown adipocytes upon acute activation *in vitro* has previously been reported [50,51], the effects of physiological activation by prolonged cold exposure on the BAT secretome remain obscure. Here, we report the transcriptomic profiles of brown and white adipocytes upon chronic cold and filtered for predicted secreted factors. This revealed CXCL12 as one of the top cold-induced secreted factors uniquely regulated and secreted from brown adipocytes upon cold activation, likely to support BAT homeostasis and optimize the thermogenic response via intercellular cross-talk with surrounding cells including brown adipocytes, neuronal cells and macrophages.

The expression of *Cxcl12* and other CXC chemokines are uniquely and differentially regulated by chronic cold in murine BAT, consistent with the tight coordination of a complex communication network orchestrating cellular homeostasis and composition to support BAT functions. The regulation of CXC chemokines in fat depots by environmental cues such as temperature or energy intake underscores their importance in the remodeling of this highly plastic organ. Short-term cold exposure was found to upregulate CXCL13 in BAT stromal cells, which would contribute to increasing thermogenesis in obese mice [52]. In the chronic cold acclimation setting, we found *Cxcl12* as the most regulated CXC chemokine in brown adipocytes, which was not observed for *Cxcl13*, nor *Cxcl14*, a previously reported batokine [8], at this time point. This likely indicates different roles for the three chemokines at different stages of BAT adaptation to cold, and in different cell populations. *Cxcl14* was shown to be primarily induced after acute cold exposure, while we observed a gradual increase of *Cxcl12* expression during 21 days of cooling, suggesting a role for CXCL12 in the long-term acclimation of BAT to cold, akin to its function in muscle adaptation to long-term exercise [53]. Additionally, cold exposure increases the secretion of CXCL12 from BAT but does not elevate its levels in plasma, underlining different roles for brown adipocyte-secreted CXCL12 and CXCL14, as CXCL14 was elegantly shown to reach the bloodstream and recruit M2 macrophages to impact iWAT browning. In contrast, CXCL12 may perform local actions in BAT by modulating the activity of adjacent cells, similar to its role in cardiac tissue [54,55]. Nevertheless, whether BAT release of CXCL12 could influence distal tissues such as cardiac muscle via direct circulatory draining, requires further exploration. Interestingly, the cold regulation of *Cxcl12* was specific to BAT, and did not occur in the other adipose depots examined, where CXCL12 has been associated with WAT inflammation and insulin resistance in mice [20,21]. By contrast, human studies reported decreased *CXCL1*2 mRNA levels with obesity that reverse after bariatric surgery-induced weight loss [56]. Albeit contradictive, this evidence may point to associations between CXCL12-adipose functions and tissue adaptation to varying conditions that are relevant to both mice and humans. CXCL14, on the other hand, has been negatively associated with obesity and type 2 diabetes [57], further strengthening the differences among these two chemokines in metabolic homeostasis. CXCL12 was found to be secreted from active human brown adipocytes [50], suggesting conserved functions across species.

Fractionation and FISH experiments indicated an enrichment of *Cxcl1*2 mRNA in brown adipocytes, especially after cold-acclimation, suggesting brown adipocytes to be the main source of CXCL12 in cold-activated BAT. Our findings are consistent with another study reporting CXCL12 among the top secreted molecules from genetically induced thermogenic adipocytes [7]. Interestingly, we also found smooth muscle cells (SMC) as another important source of CXCL12 in BAT, in accordance with a recent study, which further proposed a role for SMC-derived CXCL12 in maintaining the BAT local niche by supporting thermogenic functions [24]. More specifically, our data indicate *Cxcl12* enrichment in vein SMCs, both under thermoneutral and cold conditions. This is compatible with the characterization of *Cxcl12* expression in BAT in the Lee et al. study, which was performed at room temperature. However, the highest abundance of *Cxcl12* transcripts in brown adipocytes occurred in severe cold conditions (5 °C, maximum BAT activity). Collectively, these results imply that different pools of cells supply the necessary CXCL12 for BAT homeostasis and optimal performance, wherein brown adipocytes contribute an important regulatory function, adjusting CXCL12 release during cold adaptation.

Once released in the extracellular mileu, our results suggest pleiotropic functions for CXCL12 in the tissue, where communications between different cell types are crucial for a coordinated response. For example, paracrine signaling involving CXCL5 secreted from M1 macrophages to regulate thermogenic adipocytes was described in WAT during cold conditions [58]. Here, we report that CXCL12 produced by brown adipocytes affects chemotaxis, exerting a chemo-attractive force that may be necessary to recruit or retain macrophages in the remodeling BAT. Additionally, given the potential role of CXCL12 to guide macrophage determination [24] and the specific roles of macrophage subsets in regulating BAT function [15,59,60], it would be interesting to determine whether macrophage polarization in BAT may be influenced by brown adipocytes through CXCL12, similarly to white adipocytes [20]. In particular, whether CXCL12 may be involved in the regulation of sympathetic neuron-associated macrophages [60] to fine tune NE clearance is an intriguing speculation requiring further investigation. Consistent with the wide distribution of CXCR4 and CXCR7 across BAT, it is likely that CXCL12 facilitates cross-talk between brown adipocytes and other cell types in BAT, including with the neuronal cells terminations, which our study supports [61]. *Cxcr7* was notably found to be the most abundant G protein-coupled receptor (GPCR) in BAT [62]; thus, the use of mouse genetics will be necessary to elucidate the cellular targets of CXCL12 in BAT and decipher the complex interplay between CXCR4 and CXCR7 signaling to integrate CXCL12 signals in BAT.

Among the local effects of CXCL12 in BAT, our results suggest its requirement to directly support brown adipocyte thermogenesis. Indeed, while CXCL12 is dispensable for brown adipocytes at basal state, mitochondrial performance and the response to NE stimulation was attenuated in brown adipocytes lacking *Cxcl12*. This may be due to tampering with β-adrenergic desensitization mechanisms, as suggested by the altered gene expression pattern in cells lacking *Cxcl12* in response to NE, and involve cross-talk at the level of GPCR complex formation and internalization [63,64]. However, the involvement of other yet unclear signaling processes necessary for brown adipocyte function cannot be excluded. On the other hand, and in contrast with boosted oxygen consumption in brown adipocytes treated with exogenous administration of CXCL12 reported previously [22], enhanced production of endogenous CXCL12 was insufficient to increase the thermogenic capacity of brown adipocytes. A potential reason for the discrepancy between these results may be due to dosing and protein state (native vs. synthetic). Nevertheless, the physiological and local concentration of CXCL12 in the tissue microenvironment remains unknown and would require further exploration.

Limitations of the study. To fully understand the contribution of CXCL12 produced by brown adipocytes in the physiological adaptation of BAT to cold, further investigation using brown adipocyte specific CXCL12 gain-and-loss of function mouse models will be necessary. These will be essential to define the intercellular crosstalk mediated by CXCL12 in BAT and to determine the key cell types participating. While our study provides a deep characterization of CXCL12 regulation in BAT, it is important to consider that the work was carried out in male mice, and it would be interesting to investigate the potential influence of sex-specific hormone signals in this process and in the cold-induced transcriptomic reprogramming of adipocytes in different fat depots. Additionally, the cellular mechanism by which CXCL12 regulates thermogenic responses in brown adipocytes is still unknown, demanding further exploration in *in vivo* models.

## 5 Conclusion

A comprehensive understanding of BAT physiology is necessary to exploit its therapeutic potential to counteract the decline in metabolic health associated with obesity or ageing. Here, we provide evidence of CXCL12 production and release by brown adipocytes during cold adaptation and its potential autocrine and paracrine functions to support thermogenesis and tissue homeostasis. Further characterization of the underlying mechanisms supporting CXCL12 pleiotropic functions in BAT will help uncover the complex intercellular networks coordinating BAT remodeling and homeostasis. Furthermore, additional characterization of the role of CXCL12 and other secreted factors in BAT will advance our understanding of the adaptive mechanisms of BAT to ambient temperature, and may open new avenues to maintain or enhance BAT functions to improve metabolic health.

## CRediT authorship contribution statement

**Marina Agueda-Oyarzabal:** Writing – original draft, Investigation, Formal analysis, Conceptualization. **Marie S. Isidor:** Writing – original draft, Investigation, Formal analysis, Conceptualization. **Lars R. Ingerslev:** Formal analysis. **Oksana Dmytriyeva:** Investigation, Formal analysis. **Patricia S.S. Petersen:** Investigation, Formal analysis. **Sara Laftih:** Investigation, Formal analysis. **Axel B. Pontoppidan:** Investigation, Formal analysis. **Jo B. Henningsen:** Investigation, Formal analysis. **Kaja Rupar:** Investigation, Formal analysis. **Erin L. Brown:** Investigation, Formal analysis. **Thue W. Schwartz:** Writing – review & editing, Formal analysis. **Romain Barrès:** Writing – review & editing, Formal analysis. **Zachary Gerhart-Hines:** Writing – review & editing, Formal analysis. **Camilla C. Schéele:** Writing – review & editing, Formal analysis. **Brice Emanuelli:** Writing – original draft, Supervision, Funding acquisition, Formal analysis, Conceptualization.

## Declaration of competing interest

The authors declare the following financial interests/personal relationships which may be considered as potential competing interests: Thue W. Schwartz, Zacchary Gerhart-Hines reports a relationship with Embark Biotech ApS that includes: employment. If there are other authors, they declare that they have no known competing financial interests or personal relationships that could have appeared to influence the work reported in this paper.

## Data Availability

The original contributions presented in the study are included in the article/supplementary material; further inquiries can be directed to the corresponding author.
