# Supplementary material for: Transcriptomic signatures of cold acclimated adipocytes reveal CXCL12 as a Brown autocrine and paracrine chemokine
Source: Mol Metab. 2025 Jan 21;93:102102. doi: 10.1016/j.molmet.2025.102102 (PMC11841078; doi:10.1016/j.molmet.2025.102102)
Supplement: Multimedia component 1 [file mmc1.docx]

**Supplemental information**

| Gene | Forward primer | Reverse primer |
| --- | --- | --- |
| *Adipoq* | GATGGCACTCCTGGAGAGAA | TCTCCAGGCTCTCCTTTCCT |
| *Alpl* | CCAACTCTTTTGTGCCAGAGA | GGCTACATTGGTGTTGAGCTTTT |
| *Arg-1* | GACCAGGTGCTAATCCCTAC | CTGGTGCATCTTTGTCTCCT |
| *Atgl* | TCGTGGATGTTGGTGGAGCT | TGTGGCCTCATTCCTCCTAC |
| *Cd68* | CGGTGGAATACAATGTGTCCT | GATGAATTCTGCGCCATGA |
| *Cd206* | TCCAGTTGTTGAGGTGTTCA | GAGTGATGGTTCTCCCGTTT |
| *Ckb* | GCCTCACTCAGATCGAAACTC | GGCATGTGAGGATGTAGCCC |
| *Cox6a1* | GCACGAAGAGCACGAGAGAC | GGGTTGTGGAAGAGGGTATGG |
| *Cpt2* | CAGCACAGCATCGTACCCA | TCCCAATGCCGTTCTCAAAAT |
| *Cxcl12* | TGCATCAGTGACGGTAAACCA | TTCTTCAGCCGTGCAACAATC |
| *Cyclophilin* | CATACAGGTCCTGGCATCTTGTC | AGACCAACATGCTTGCCATCCAG |
| *Dgat1* | GAGGCCTCTCTGCCCCTATG | GCCCCTGGACAACACAGACT |
| *Dgat2* | CCGCAAAGGCTTTGTGAAG | GGAATAAGTGGGAACCAGATCA |
| *Eci1* | ACTGGAAGAACGTGCAGGAG | AAACCAGAAGGGGGCAACAA |
| *Fabp4* | ACAGGAAGGTGAAGAGCATC | CCTTTGGCTCATGCCCTTTC |
| *Fasn* | ATTGGTGGTGTGGACATGGTC | CCCAGCCTTCCATCTCCTG |
| *Gk* | AACACGGGCCATAAGTGTGT | TAGCCACGGAACCTTCCAAC |
| *Glut1* | GGGCTGCCAGGTTCTAGTC | CCTCCGAGGTCCTTCTCA |
| *Hsl* | CACCCATAGTCAAGAACCCCTTC | TCTACCACTTTCAGCGTCACCG |
| *Il6* | GAGGATACCACTCCCAACAGACC | AAGTGCATCATCGTTGTTCATACA |
| *IL10* | AGCTCCAAGACCAAGGTGTC | TCCAAGGAGTTGTTTCCGTTA |
| *iNOS* | AGAGAACGGAGAACGGAGAA | GCACAAGGGGTTTTCTTCAC |
| *leptin* | ATGTGCCCTTCCGATATACAACC | CGTGTCATCCACTAATCTTCTGG |
| *Mgl* | CGGACTTCCAAGTTTTTGTCAGA | GCAGCCACTAGGATGGAGATG |
| *Pgc1α* | TGATGTGAATGACTTGGATACAGACA | GCTCATTGTTGTACTGGTTGGATAT |
| *Pmp70* | GGCCTGCACGGTAAGAAAAGT | CCGCAATAAGTAACAAGTAGCCT |
| *RyR1* | CCAGTCGCTGCCTCTCAC | GCAGCCTCTTGTTGTCATCC |
| *RyR2* | CTGAGTGCAGTTGCGGGAG | AGGTTGTGGGTTAAAGTTCCCT |
| *Serca1* | GAACCTTTGCCGCTCATTTT | AGGCTGCACACACTCTTTACC |
| *Serca2* | ATGGCAATTGGGGGCTATGT | GGCCTCAAAGACCTCACAGT |
| *Tbp* | ACCCTTCACCAATGACTCCTATG | TGACTGCAGCAAATCGCTTGG |
| *TNFα* | TCTTCTCATTCCTGCTTGTGG | GGTCTGGGCCATAGAACTGA |
| *Vegf* | CTTGTTCAGAGCGGAGAAAGC | ACATCTGCAAGTACGTTCGTT |
| *Ucp1* | AGCCGGCTTAATGACTGGAG | TCTGTAGGCCTGCCCAATGAAC |

**Table S1.** RT-qPCR primer sequences.

| Antibody | Dilution | Cat. No.  (Cell Signaling) |
| --- | --- | --- |
| Vinculin e1e9v xp rabbit mab | 1:2000 | #13901 |
| P-CREB (ser133) (87G3) rabbit mab | 1:2000 | #9198 |
| P-P38 MAPK (thr189/tyr182) D3f9 XP rabbit mab | 1:2000 | #4511 |
| p-PKA substrate (RRXS*/T*) rabbit mab | 1:2000 | #9624 |

**Table S2:** Antibody dilutions and catalogue numbers for immunoblotting.

**Table S3.** Differential gene expression dataset (related to Figure 1 & 2). The Excel Workbook contains 8 sheets with the results of differential gene expression (DGE) analysis. The first sheet called “Instructions” contains the details, explanation of contrasts, and acronyms for each comparison. The remaining data sheets only contain the results of DGE analysis.


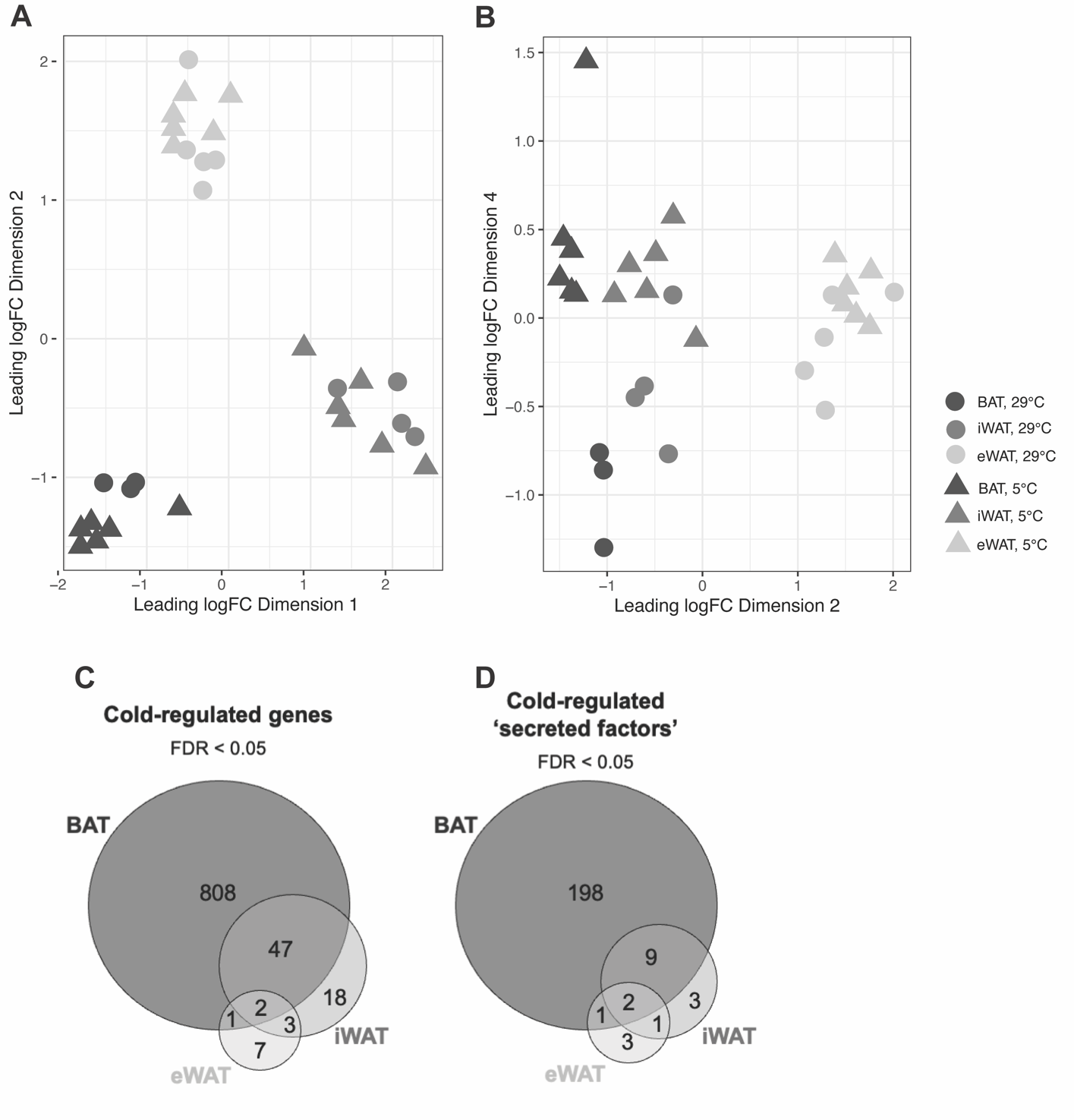


**Figure S1.** **A.** Principal component analysis (PCA) plot of dimensions 1 and 2 of gene expression in adipocytes from BAT, iWAT and eWAT of mice exposed to thermoneutrality (29°C) and cold conditions (5°C). **B.** PCA plot of dimensions 3 and 4 of gene expression in adipocytes from BAT, iWAT and eWAT of mice exposed to thermoneutrality (29°C) and cold conditions (5°C). **C.** Venn diagram of all differentially regulated genes (FDR < 0.05). **D**. Venn diagram of all differentially regulated genes predicted to encode secreted factors (FDR < 0.05).


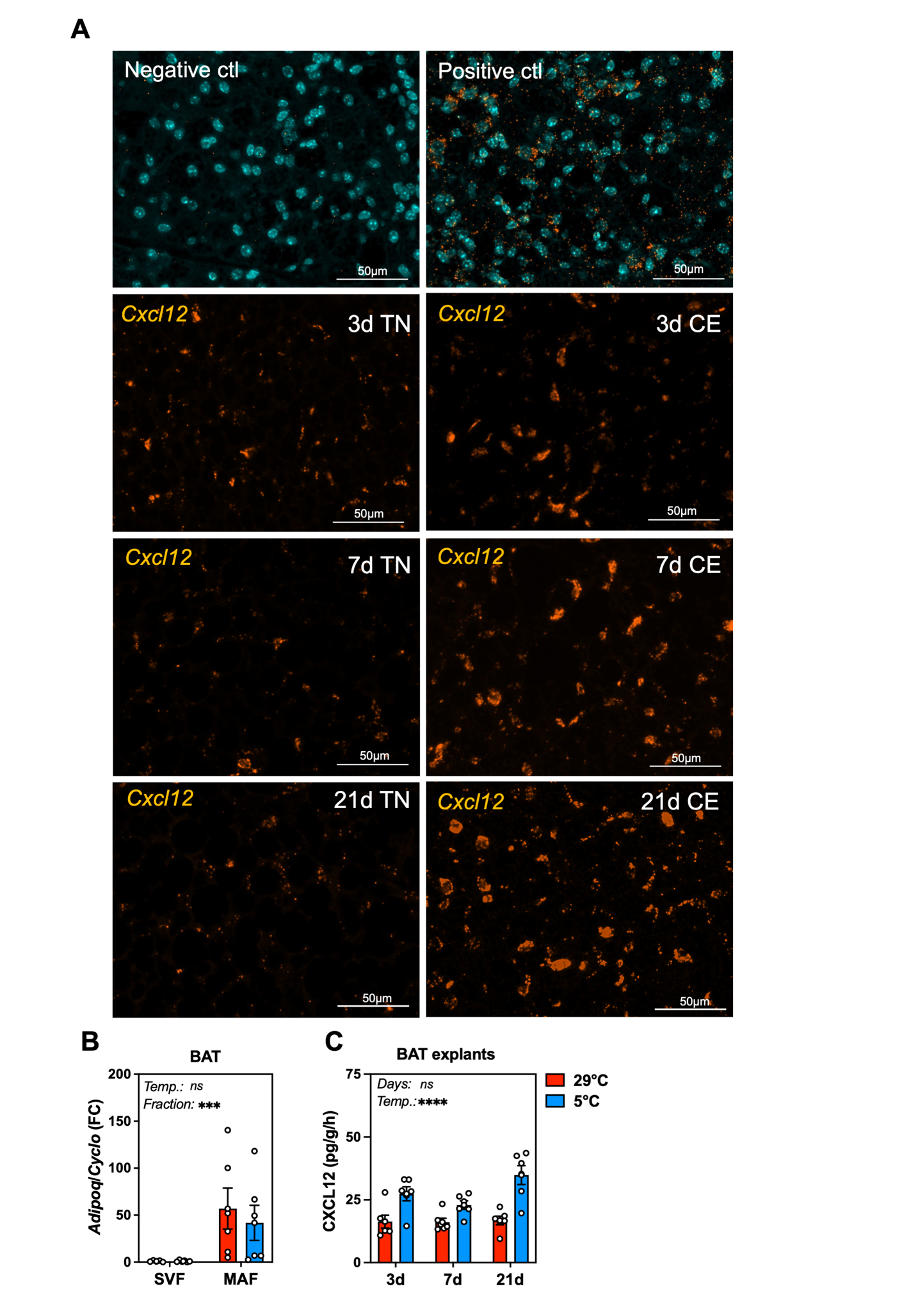


**Figure S2. A.** FISH using a negative (Negative ctl) or positive (Positive ctl) control probe (orange) with DAPI staining (blue) in BAT (representative image) (n=3). *Cxcl12* FISH in BAT, 3 days, 7 days or 21 days following housing at thermoneutrality (3d, 7d, or 21d TN) or in the cold (3d, 7d, or 21d CE) (representative image) (n=3). **B.** mRNA expression of *Adipoq* in the stromal vascular fraction (SVF) and mature adipocyte fraction (MAF) isolated from BAT of mice exposed to thermoneutrality (29°C) and cold conditions (5°C) (n=6). **C.** Secretion rate of CXCL12 by BAT explants at thermoneutrality (29°C) and after activation by 3, 7 and 21 days of cold exposure (5°C) (n=6). Statistical significance was performed using a 2-way ANOVA (α<0.05). (*) p<0.05; (***) p<0.001; (****) p<0.0001.


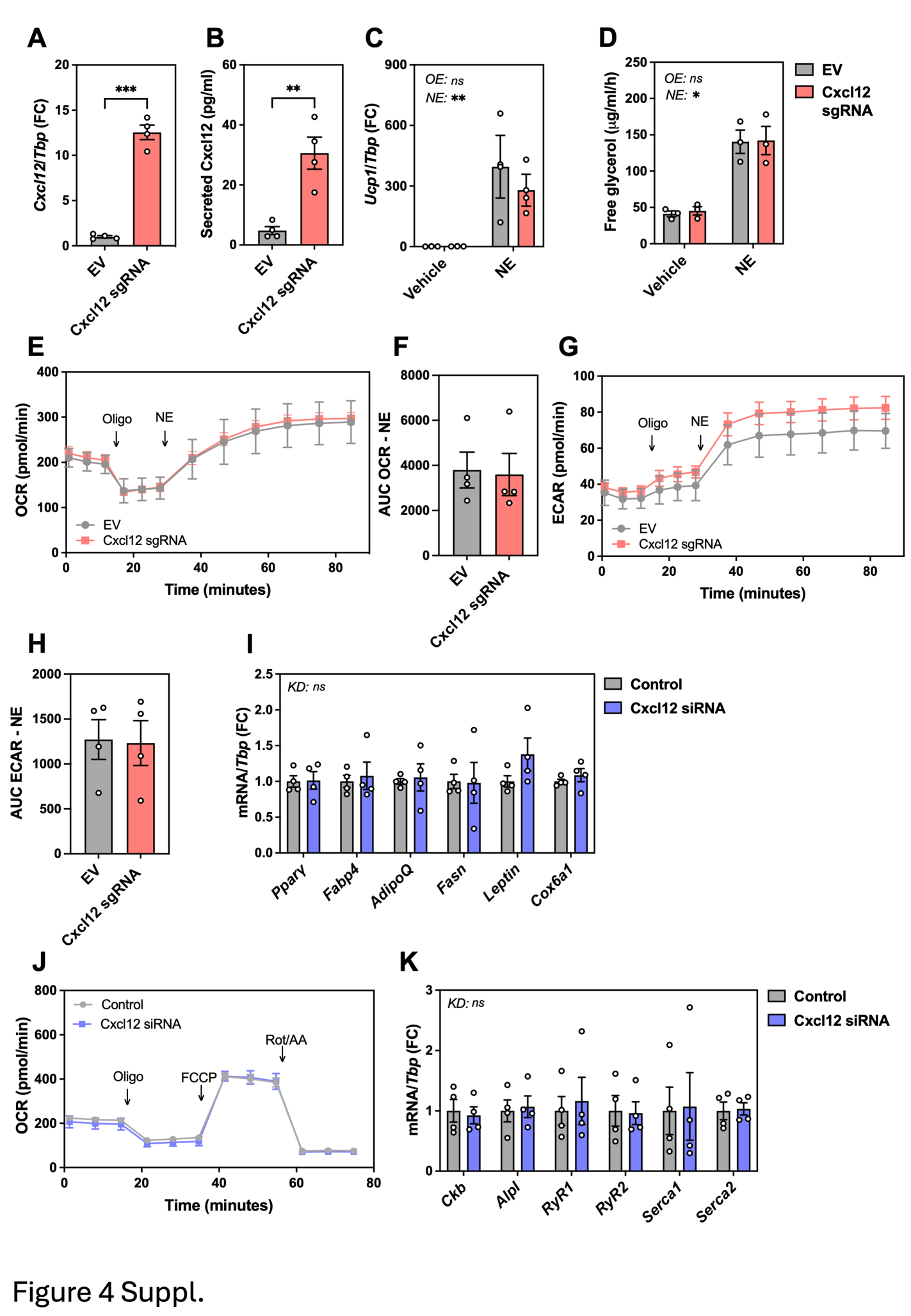


**Figure S3.** **A-B.** CRISPRa induced *Cxcl12* expression **(A)** and CXCL12 secretion **(B)** in murine mature brown adipocytes (n=4). EV: empty vector; Cxcl12 sgRNA: Cxcl12 single-guide RNA**. C.** *Ucp1* mRNA expression in control and *Cxcl12* overexpressing brown adipocytes after NE stimulation (n=3). **D.** NE-stimulated glycerol release in control and *Cxcl12* overexpressing brown adipocytes (n=3). **E-H.** Mitochondrial respiration in response to NE in control and *Cxcl12* overexpressing murine mature brown adipocytes, **(E)** OCR from representative experiment and **(F)** OCR quantification (n=4), **(G)** ECAR from representative experiment and **(H)** ECAR quantification (n=4). **I.** mRNA expression of selected genes in control and *Cxcl12* KD murine mature brown adipocytes (n=4). **J.** Mitochondrial stress test in control and *Cxcl12* KD murine mature brown adipocytes, representative experiment (n=3). **K.** mRNA expression of selected genes in control and *Cxcl12* KD murine mature brown adipocytes (n=4). Statistical significance was performed using a t-test (paired, two-tailed) (p<0.05) or a 2-way ANOVA (α<0.05). (*) p<0.05; (**) p<0.01; (***) p<0.001.


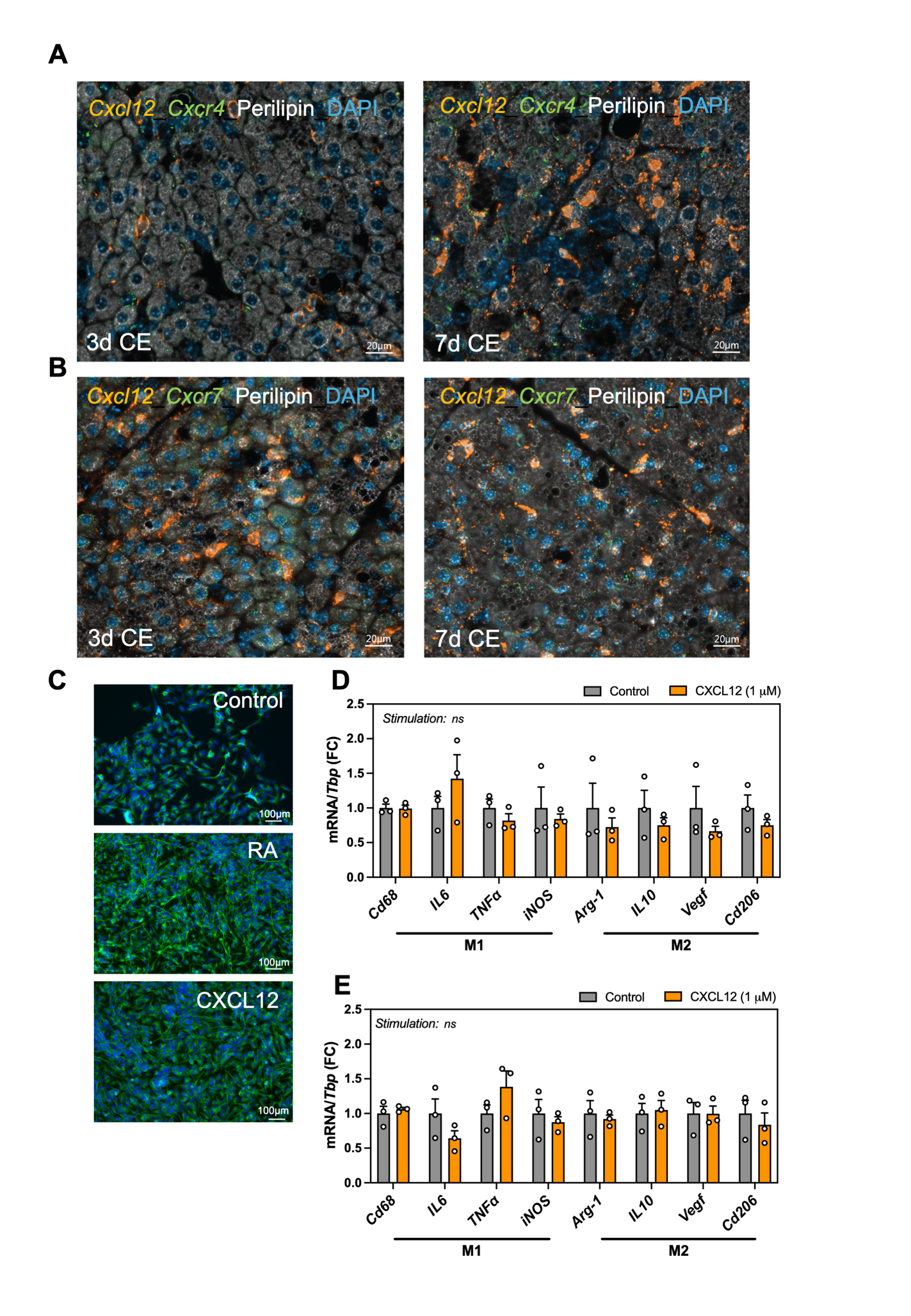


**Figure S4. A-B.** FISH for (**A**) *Cxcr4* or (**B**) *Cxcr7* (green), *Cxcl12* (orange) and immunohistochemistry staining of perilipin (white) and DAPI (blue) in BAT following 3 days **(left)** and 7 days **(right)** of cold exposure (5°C) (representative images, n=3). **C.** Representative images of average neurite outgrowth of SH-SY5Y neurons treated with retinoic acid (RA) or recombinant human CXCL12 (0.1 μM CXCL12). **D-E.** mRNA expression of selected genes in upon stimulation of macrophages with 1 μM CXCL12 for 3 hours (**D**) or 6 hours (**E**).
